# Immunomodulatory Effects of *Phallus indusiatus* Extract on Cytokine Responses in PBMCs: Implications for Feline Infectious Peritonitis

**DOI:** 10.3390/ijms27031437

**Published:** 2026-01-31

**Authors:** Chularat Hlaoperm, Wassamon Moyadee, Emwalee Wongsaengnoi, Lueacha Tabtimmai, Amonpun Rattanasrisomporn, Atchara Paemanee, Kiattawee Choowongkomon, Christopher Gerner, Oumaporn Rungsuriyawiboon, Jatuporn Rattanasrisomporn

**Affiliations:** 1 Graduate Program in Animal Health and Biomedical Sciences, Faculty of Veterinary Medicine, Kasetsart University, Bangkok 10900, Thailand; chularat.h@ku.th; 2 Department of Companion Animal Clinical Sciences, Faculty of Veterinary Medicine, Kasetsart University, Bangkok 10900, Thailand; wassamon.moy@ku.th; 3 Department of Veterinary Technology, Faculty of Veterinary Technology, Kasetsart University, Bangkok 10900, Thailand; emwalee.wo@ku.th (E.W.); cvtopr@ku.ac.th (O.R.); 4 Department of Biotechnology, Faculty of Applied Science, King Mongkut’s University of Technology North Bangkok, Bangkok 10800, Thailand; lueacha.t@sci.kmutnb.ac.th; 5 Food and Agro-Industrial Research Center, King Mongkut’s University of Technology North Bangkok, Bangkok 10800, Thailand; 6 Interdisciplinary of Genetic Engineering and Bioinformatics, Graduate School, Kasetsart University, Bangkok 10900, Thailand; fgraapr@ku.ac.th; 7 National Omics Center, National Center for Genetic Engineering and Biotechnology (BIOTEC), National Science and Technology Development Agency (NSTDA), Khlong Luang, Pathum Thani 12120, Thailand; atchara.pae@biotec.or.th; 8 Food Biotechnology Research Team, Functional Ingredients and Food Innovation Research Group, National Center for Genetic Engineering and Biotechnology (BIOTEC), National Science and Technology Development Agency (NSTDA), Khlong Luang, Pathum Thani 12120, Thailand; 9 Department of Biochemistry, Faculty of Science, Kasetsart University, Bangkok 10900, Thailand; fsciktc@ku.ac.th; 10 Faculty of Chemistry, Department of Analytical Chemistry, University of Vienna, Waehringer Strass 38, 1090 Vienna, Austria; christopher.gerner@univie.ac.at

**Keywords:** feline coronavirus, feline infectious peritonitis, *Phallus indusiatus*, anti-inflammatory, cytokines, antiviral, interferon-gamma (IFN-γ), interleukin-1β (IL-1β), interleukin-10 (IL-10)

## Abstract

Feline infectious peritonitis (FIP) is a fatal disease driven by feline coronavirus induced immune dysregulation and excessive inflammatory cytokine production. Immunomodulatory agents capable of rebalancing this response are therefore of increasing interest. *Phallus indusiatus* (*P. indusiatus*), an edible mushroom containing diverse bioactive compounds, has previously demonstrated antiviral and anti-inflammatory potential. This study investigated the immunomodulatory effects of *P. indusiatus* extract on peripheral blood mononuclear cells (PBMCs) from healthy cats and FIP cats and characterized its chemical constituents using liquid chromatography coupled with tandem mass spectrometry (LC–MS/MS). PBMCs were isolated from whole blood and FIP fluid. Cytotoxicity screening identified 19 µg/mL as a non-toxic concentration for subsequent assays. Cytokine responses (IL-1β, IFN-γ, and IL-10) were evaluated following LPS stimulation in PBMCs from whole blood and under basal conditions in PBMCs from FIP fluid after treatment with *P. indusiatus* extract and dexamethasone. LC–MS/MS profiling combined with STITCH analysis was used to identify bioactive metabolites and their predicted molecular targets. PBMCs derived from FIP fluid exhibited markedly elevated IL-1β and IFN-γ, indicating strong baseline immune activation. *P. indusiatus* significantly reduced IL-1β and IFN-γ in PBMCs from FIP fluid and suppressed LPS-induced IL-1β and IL-10 in whole-blood PBMCs, demonstrating immunomodulatory patterns comparable to dexamethasone. LC–MS/MS analysis identified compounds including adenosine, phenylalanine, tyrosine, cystathionine, arginine, and sialic acid, which were linked to inflammatory signaling. Overall, the extract exhibited context-dependent modulation of pro- and anti-inflammatory cytokines, suggesting that *P. indusiatus* may serve as a promising natural adjunctive candidate for managing immune imbalance in cats with FIP.

## 1. Introduction

Feline coronavirus (FCoV), an enveloped positive-sense RNA virus belonging to the Coronaviridae family, is highly prevalent among domestic cats worldwide [1,2]. It occurs in two biotypes: feline enteric coronavirus (FECV), which typically causes mild and self-limiting enteritis, and feline infectious peritonitis virus (FIPV), the virulent form responsible for feline infectious peritonitis (FIP) [3,4]. FCoV-infected cats develop FIP, with immune dysfunction and environmental stress identified as key predisposing factors [5]. The pathogenesis involves FCoV replication in activated monocytes and macrophages, resulting in systemic inflammation, pyogranulomatous lesions, and T-cell depletion [6,7,8]. Altered cytokine profiles, characterized by elevated levels of interleukin (IL)-1β, IL-6, IL-10, tumor necrosis factor-alpha (TNF-α) and interferon-gamma (IFN-γ), were higher in cats with FIP [9,10,11]. Although several antiviral agents (e.g., remdesivir, molnupiravir, GS-441524, and GC376) have recently become available, their clinical effectiveness remains limited due to delayed diagnosis, clinical relapse and drug resistance may occur [12,13,14,15]. Viral infections frequently exploit imbalances in immune responses, leading either to insufficient viral clearance or excessive inflammatory damage [16]. These insights underscore the urgent need for adjunctive therapies that can concurrently suppress viral replication and restore immune homeostasis while enhancing antiviral defense mechanisms [17]. Therefore, immune-modulating interventions represent a promising strategy for the development of novel therapeutic approaches against FIP [18].

Medicinal mushrooms are well known for their bioactive compounds with immunomodulatory, anti-inflammatory, and antiviral properties [19,20]. Similarly, *P. indusiatus*, an edible mushroom widely distributed in tropical regions, has shown promising potential for biomedical applications [21]. Our previous studies demonstrated that *P. indusiatus* extract inhibited feline coronavirus main protease (FIPV M^pro^) activity and reduced nitric oxide (NO) production in lipopolysaccharide (LPS)-stimulated PBMCs, suggesting both antiviral and anti-inflammatory potential [22]. Moreover, chemical profiling of *P. indusiatus* has identified bioactive molecules such as adenosine and N-acetylglucosamine (NAG), which are known to regulate cytokine secretion and promote immune balance [23,24,25].

Despite these promising findings, little is known about the direct immunomodulatory effects of *P. indusiatus* on immune cells from companion animals, particularly in the context of FIP. To address this gap, the present study aimed to evaluate the effects of *P. indusiatus* extract on cytokine responses in PBMCs derived from healthy cats and FIP-infected cats. Specifically, we investigated its influence on pro-inflammatory cytokines (IL-1β and IFN-γ) and the anti-inflammatory cytokine (IL-10). In addition, LC–MS/MS was used to characterize bioactive constituents in the extract, providing mechanistic insights into how *P. indusiatus* may contribute to antiviral and immunomodulatory strategies for FIP.

## 2. Results

### 2.1. Hematology

The hematological profiles of eight donor cats and eight cats diagnosed with effusive FIP are summarized in Table 1, alongside established hematology reference ranges [26]. Comparative analysis revealed significant differences in several hematological parameters, including hemoglobin (HGB), packed cell volume (PCV), red blood cell count (RBC), white blood cell counts (WBCs), reticulocytes (RETICs), and segmented neutrophils (Segs) (*p* < 0.05). In particular, HGB, PCV, Segs, and RBC values were significantly lower in the FIP group compared with healthy donor cats (*p* < 0.01). A total of 20 cats diagnosed with FIP were initially recruited for sample collection; however, after PBMCs isolation, only 8 cases yielded sufficient viable white blood cells for downstream cytokine assays. Many samples contained inadequate cell numbers or showed marked cell aggregation, making them unsuitable for experimentation. Therefore, the eight samples with adequate PBMCs yield and acceptable cell quality were selected as the appropriate and reliable specimens for subsequent immunological testing. In FIP cats, PCR for FIPV, Rivalta’s test, effusion characteristics, and A:G ratios were evaluated (Supplementary Table S1). All cases presented with yellow fluid, viscous effusions and an A:G ratio below 0.5, consistent with FIP cats. Effusion samples from cats with confirmed FIP were evaluated using Rivalta’s test to distinguish exudative from transudative effusions based on protein concentration and inflammatory content, together with PCR confirmation. FIP fluid samples from the eight affected cats were subsequently used to isolate PBMCs.

### 2.2. Effect of P. indusiatus on the Cell Viability of PBMCs

**Figure 1:**
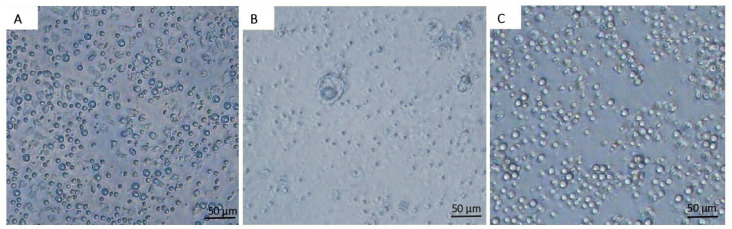
Morphological effects of *P. indusiatus* extract on PBMCs from whole blood (1 × 10^6^ cells/mL) observed under an inverted microscope. (Scale bar = 50 µm.) Untreated control PBMCs (**A**). PBMCs were treated with 5000 µg/mL of *P. indusiatus* extract, showing cytotoxic effects (**B**). PBMCs were treated with 19 µg/mL of *P. indusiatus* extract, retaining normal morphology (**C**).

The morphology of PBMCs from whole blood was examined after 24 h exposure to various concentrations of *P. indusiatus* extract using the CCK-8 assay. In the control group (Figure 1A), cells retained a spherical shape. Concentrations from 10,000 to 39 µg/mL induced morphological changes, including reduced cell size, loss of sheen, and cell death, accompanied by viability below 90% (Figure 1B). At 19 µg/mL, PBMCs maintained normal morphology and viability exceeded 90%, comparable to the control (Figure 1C).

**Figure 2:**
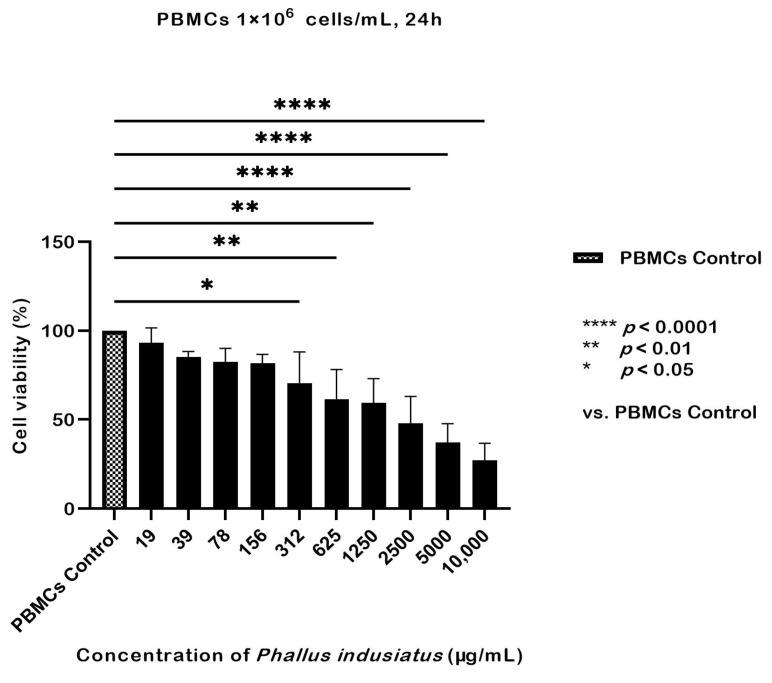
PBMC viability was tested with *P. indusiatus* extract at various concentrations, and a concentration of 19 µg/mL was selected for subsequent testing (mean ± SEM).

The toxicity of *P. indusiatus* extract at various concentrations on PBMCs from whole blood was assessed over 24 h using the CCK-8 colorimetric assay. At 10,000, 5000, 2500, 625, and 312 µg/mL, cell viability dropped below 90%, indicating that these concentrations are unsuitable for further testing as they may affect cytokine secretion. Cell viability was significantly reduced compared with the control *(p* < 0.05); however, at concentrations of 156, 78, and 39 µg/mL, viability remained at approximately 80% and did not differ significantly from the control *(p >* 0.05). At 19 µg/mL, cell viability remained close to 90% and was not significantly different from the control indicating that this concentration is suitable for further testing (Figure 2).

**Figure 3:**
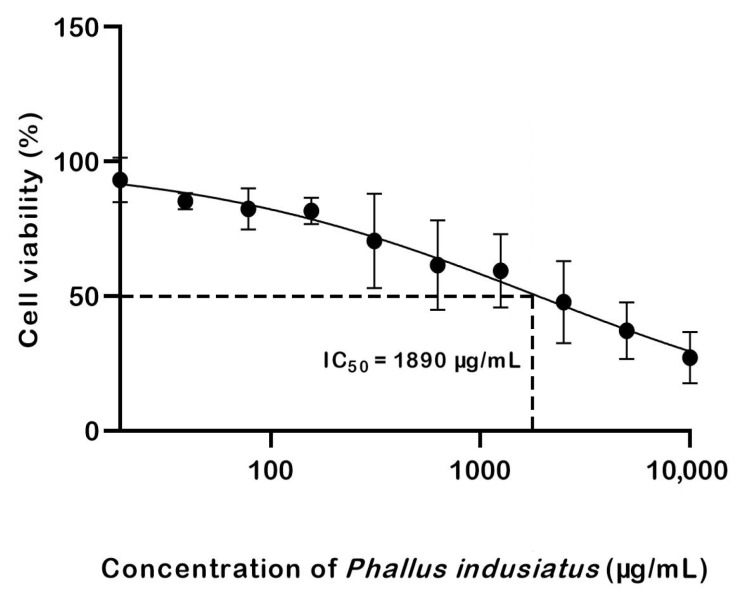
PBMCs were exposed to increasing concentrations of *P. indusiatus* extract (19–10,000 µg/mL) for 24 h, and cell viability was assessed. Data are presented as mean ± SEM. The dashed lines indicate the IC_50_ value (1890 µg/mL).

The IC_50_ value of *P. indusiatus* was calculated to be 1890 µg/mL, as shown in Figure 3.

### 2.3. Cytokine Levels in PBMCs After LPS Stimulation

**Figure 4:**
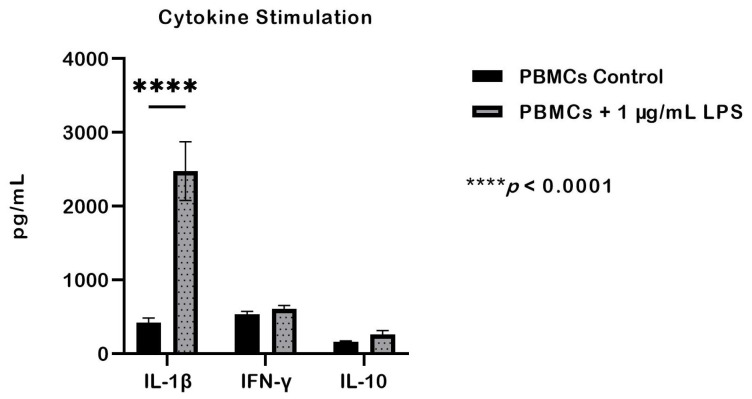
Cytokine levels in PBMCs stimulated with 1 µg/mL LPS compared with unstimulated controls. LPS stimulation significantly increased IL-1β production (*p* < 0.05), whereas no statistically significant differences were observed in IFN-γ or IL-10 levels. Data are presented as mean ± SEM and were analyzed by one-way ANOVA, with *p* < 0.05 considered statistically significant (*n* = 3).

The levels of proinflammatory cytokines (IL-1β and IFN-γ) and the anti-inflammatory cytokine (IL-10) were quantified after stimulation. LPS stimulation significantly increased IL-1β levels compared with the unstimulated control group (*p* < 0.05), whereas, IFN-γ levels did not differ significantly between groups. Similarly, IL-10 levels were not significantly different from the control, although a numerical increase was observed, rising from 165.39 pg/mL to 265.58 pg/mL (Figure 4).

### 2.4. Enhanced Cytokine Production in PBMCs from FIP Fluid Compared with Whole Blood

**Figure 5:**
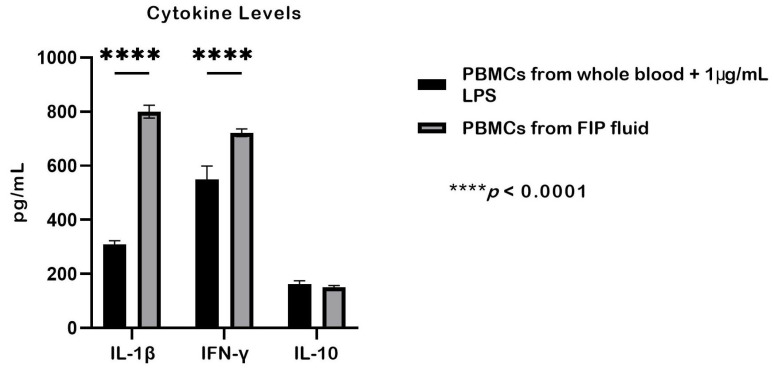
Cytokine production in PBMCs from whole blood stimulated with 1 µg/mL LPS compared with PBMCs isolated from FIP fluid without stimulation. IL-1β and IFN-γ levels were significantly higher in PBMCs from FIP fluid than in LPS-stimulated PBMCs from whole blood (*p* < 0.0001), whereas IL-10 levels did not differ significantly between the two groups. Data are presented as mean ± SEM and were analyzed by two-way ANOVA, with *p* < 0.05 considered statistically significant (*n* = 3).

PBMCs derived from FIP fluid showed higher cytokine production compared with LPS-stimulated PBMCs from whole blood (Figure 5). IL-1β levels were significantly higher in PBMCs from FIP fluid (800 ± 23 pg/mL) than in LPS-stimulated PBMCs from whole blood (308 ± 14 pg/mL; *p* < 0.0001). Similarly, IFN-γ levels were markedly elevated in PBMCs from FIP fluid (722 ± 13 pg/mL) compared with LPS-stimulated PBMCs from whole blood (549 ± 49 pg/mL; *p* < 0.0001). In contrast, IL-10 levels did not differ significantly between PBMCs from FIP fluid (150 ± 6 pg/mL) and LPS-stimulated PBMCs from whole blood (162 ± 10 pg/mL). These findings indicate that PBMCs from FIP fluid exhibit spontaneous cytokine release even in the absence of LPS stimulation [22,27].

### 2.5. Modulation of Pro-Inflammatory and Anti-Inflammatory Cytokines in LPS-Stimulated and FIP-Derived PBMCs by P. indusiatus

This study assessed the immunomodulatory effects of *P. indusiatus* by measuring cytokine levels in PBMCs from whole blood following LPS stimulation and PBMCs from FIP fluid. The effects of *P. indusiatus* extract were compared with the anti-inflammatory drug dexamethasone as a positive control. Specifically, the influence of *P. indusiatus* on pro-inflammatory cytokines (IFN-γ and IL-1β) and the anti-inflammatory cytokine (IL-10) was examined in PBMCs from both whole blood and FIP fluid.

**Figure 6:**
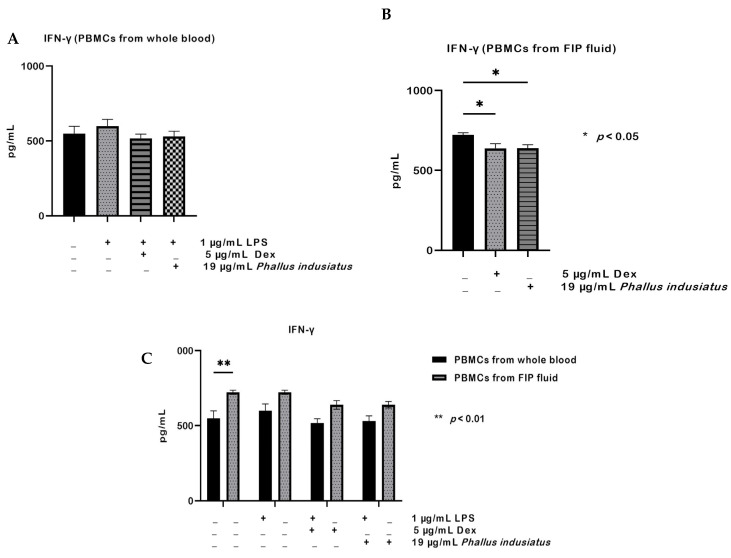
IFN-γ production in PBMCs from whole blood and FIP fluid. Cells were stimulated with 1 µg/mL LPS and treated with 5 µg/mL dexamethasone (DEX) and 19 µg/mL *P. indusiatus* extract (**A**). IFN-γ levels in whole-blood PBMCs showed no significant changes (**B**). In PBMCs from FIP fluid, dexamethasone and *P. indusiatus* significantly reduced IFN-γ compared with the untreated group (**C**). PBMCs from FIP fluid consistently produced higher IFN-γ than PBMCs from whole blood. Data are mean ± SEM (*n* = 3); statistical significance was assessed by one- or two-way ANOVA (* *p* < 0.05; ** *p* < 0.01).

For IFN-γ, PBMCs from whole blood showed no significant changes after LPS stimulation or treatment with dexamethasone or *P. indusiatus* (Figure 6A). In contrast, IFN-γ levels in PBMCs from FIP fluid were significantly reduced by both dexamethasone and *P. indusiatus* compared with the untreated group (*p* < 0.05; Figure 6B). When comparing the two cell sources, PBMCs from FIP fluid consistently produced significantly higher IFN-γ levels than PBMCs from whole blood *p* < 0.01; Figure 6C). The anti-inflammatory agents dexamethasone and *P. indusiatus* showed no statistically significant effect on IFN-γ levels in either cell group.

**Figure 7:**
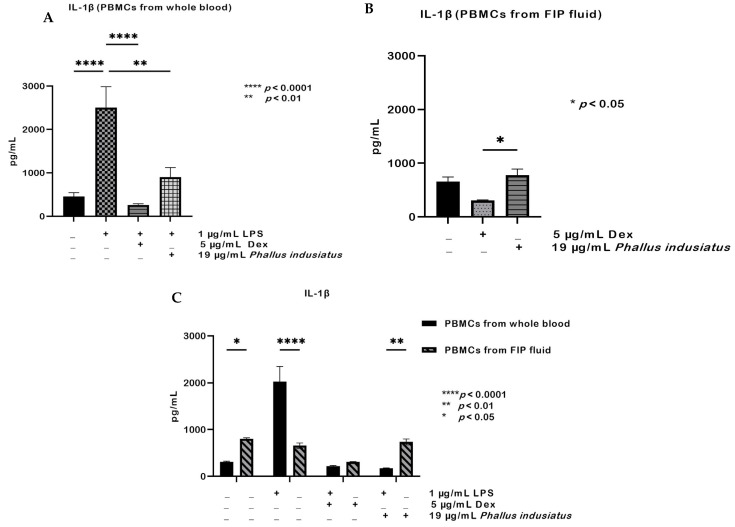
IL-1β production in PBMCs from whole blood and PBMCs from FIP fluid under 1 µg/mL LPS stimulation and treatment with 5 µg/mL dexamethasone and 19 µg/mL *P. indusiatus* (**A**). LPS significantly increased IL-1β in PBMCs from whole blood, while both treatments suppressed this effect (**B**). In PBMCs from FIP fluid, dexamethasone significantly reduced IL-1β levels compared with the untreated group, whereas *P. indusiatus* treatment showed no statistically significant difference despite a slightly higher mean value (**C**). PBMCs from FIP fluid produced higher IL-1β without stimulation, whereas LPS induced higher IL-1β in PBMCs from whole blood. Data are mean ± SEM (*n* = 3); statistical significance was assessed by one- or two-way ANOVA (* *p* < 0.05; ** *p* < 0.01; **** *p* < 0.0001).

A similar trend was observed for IL-1β. In PBMCs from whole blood, LPS stimulation markedly increased IL-1β production compared with the control group (*** *p* < 0.0001), whereas dexamethasone or *P. indusiatus* significantly suppressed this effect (* *p* < 0.01; Figure 7A). When comparing the anti-inflammatory effect of dexamethasone with that of the tested extract, no statistically significant difference was found. In PBMCs from FIP fluid, IL-1β levels were also reduced by dexamethasone and *P. indusiatus* extracts relative to the untreated group (*p* < 0.05; Figure 7B). Although the mean IL-1β level in the *P. indusiatus*–treated group appeared slightly higher than that in the untreated group, this difference was not statistically significant and likely reflects inter-individual variability rather than a true biological effect. When comparing the anti-inflammatory activity of dexamethasone with that of the tested extracts, dexamethasone was found to reduce IL-1β levels significantly more than the extracts. Comparison between the two cell sources revealed that PBMCs from FIP fluid produced higher IL-1β than PBMCs from whole blood in the absence of stimulation. However, upon LPS stimulation, IL-1β production was significantly greater in PBMCs from whole blood than in PBMCs from FIP fluid. Under dexamethasone treatment, no significant differences were observed between the two groups, whereas treatment with *P. indusiatus* resulted in a statistically significant difference. Although IL-1β levels were reduced in both cell types compared with the untreated group, PBMCs from FIP fluid still produced higher levels than PBMCs from whole blood (Figure 7C).

**Figure 8:**
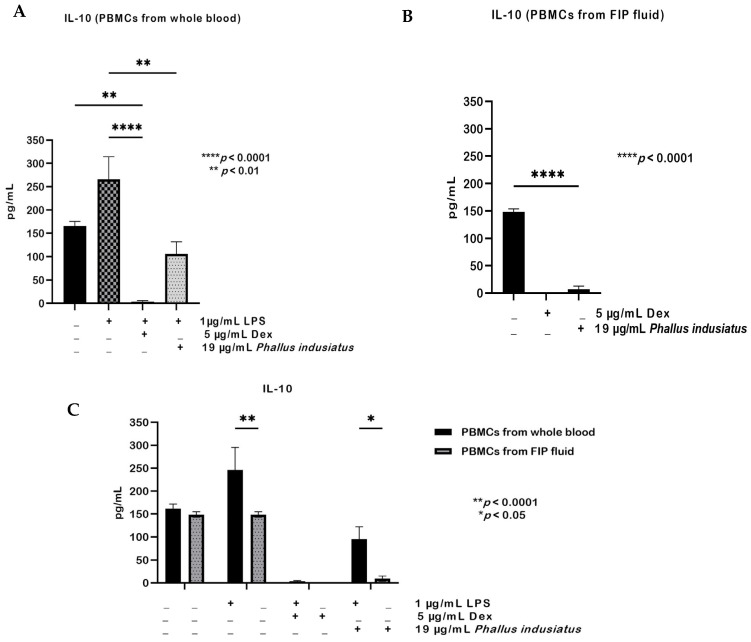
IL-10 production in PBMCs from whole blood after 1 µg/mL LPS stimulation and PBMCs from FIP fluid, treated with 5 µg/mL dexamethasone (Dex) and *P. indusiatus* extract (19 µg/mL) (**A**). In PBMCs from whole blood, LPS markedly increased IL-10, which was suppressed by both treatments (**B**). In PBMCs from FIP fluid, IL-10 production was generally low and further reduced by treatments (**C**). IL-10 induction was robust in PBMCs from whole blood but markedly impaired in PBMCs from FIP fluid. Data are mean ± SEM (*n* = 3); statistical significance was assessed by one- or two-way ANOVA (* *p* < 0.05; ** *p* < 0.01; **** *p* < 0.0001).

For IL-10, LPS stimulation markedly increased production in PBMCs from whole blood, whereas both dexamethasone and *P. indusiatus* significantly suppressed this induction (Figure 8A). In contrast, PBMCs from FIP fluid exhibited overall impaired IL-10 production, and treatment with either agent further reduced cytokine levels compared with the untreated group (Figure 8B). Comparison between the two cell sources revealed robust IL-10 induction in PBMCs from whole blood following LPS stimulation, which was significantly higher than in PBMCs from FIP fluid. Both cell types showed a comparable suppressive response to dexamethasone, while *P. indusiatus* caused a greater reduction in IL-10 levels in PBMCs from FIP fluid than in PBMCs from whole blood, with statistical significance (Figure 8C).

**Figure 9:**
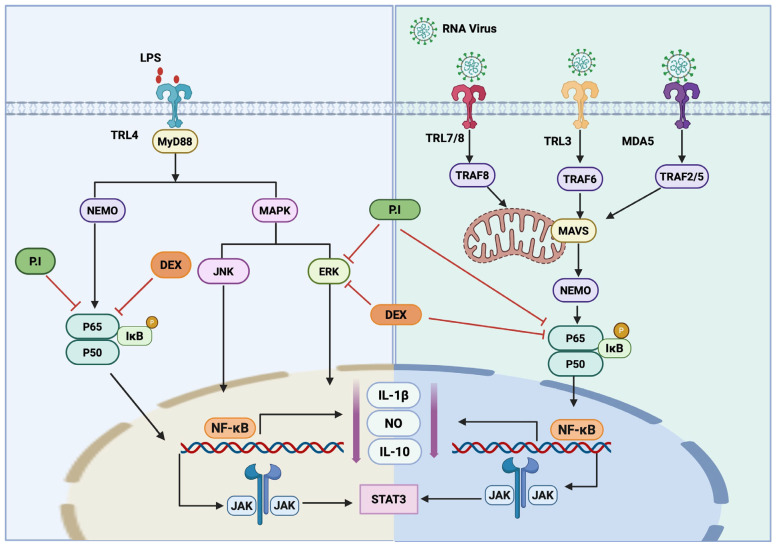
Signaling pathway showing the modulatory effects of *P. indusiatus* (P.I) extract and dexamethasone (DEX) on inflammatory activation illustrates the upstream receptor-mediated inflammatory pathways activated by LPS or RNA viruses and highlights how *P. indusiatus* extract and dexamethasone (DEX) differentially modulate these signaling cascades.

Comparative signaling pathway showing receptor-level activation and inhibition by *P. indusiatus* extract and dexamethasone. The signaling framework contextualizes the cytokine responses observed in this study. In LPS-stimulated PBMCs from whole blood, both *P. indusiatus* and dexamethasone reduced IL-1β and IL-10 secretion, while IFN-γ remained unchanged. In PBMCs isolated from FIP fluid, which exhibited elevated basal IL-1β and IFN-γ levels, both treatments suppressed these cytokines, with *P. indusiatus* producing a stronger reduction in IL-10. The figure provides an integrated pathway layout that corresponds to the observed modulation of inflammatory cytokines under LPS and viral-associated receptor stimulation. Consistent across both conditions, reduced nuclear factor kappa B (NF-κB) activity was associated with decreased expression of IL-1β and NO, together with reduced IL-10 expression (Figure 9).

### 2.6. Bioactive Compounds

LC–MS/MS multiple bioactive constituents in the *P. indusiatus* extract (Table 2). The detected compounds included amino acids (L-histidine, L-phenylalanine, L-tyrosine), nucleosides (adenosine), sugar derivatives (mannitol) and acetylated sugars (NAG, N-acetylgalactosamine; GalNAc). Additional minor components such as cystathionine and salialic acid were also detected. Several of these molecules have been previously reported to exert anti-inflammatory or immunomodulatory effects [23,24,28,29].

**Figure 10:**
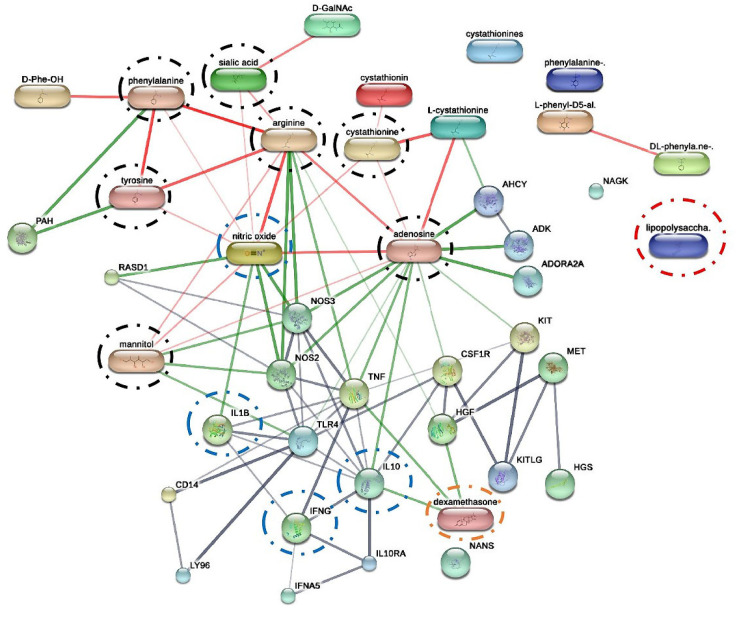
STITCH 5.0 network showing interactions between *P. indusiatus* bioactive compounds and inflammatory targets. Most compounds (adenosine, phenylalanine, and tyrosine) were connected to IL-1β (IL1), IFN-γ (IFNG) (green and gray lines), NOS_2_ and NOS_3_ (red lines), and IL-10 (green lines). These predicted interactions align with the observed suppression of pro-inflammatory cytokines and the increase in IL-10 (Table 2). Dexamethasone (positive control) showed similar predicted interactions (green lines).

To explore potential molecular interaction, a protein-chemical association network was generated using the STITCH 5.0 database for *Felis catus* (Figure 10). The analysis integrated *P. indusiatus-derived* compounds with known inflammatory mediators and reference compound including dexamethasone. The resulting network displayed multiple associations among identified metabolites, cytokine, and signals protein. Notably, adenosine, arginine, and phenylalanine appeared as major nodes connected to nitric oxide synthase (NOS_2_, NOS_3_), IL-10, TNF, IL1-β, and IFN-γ, suggesting their relevance to immune-related pathways.

Summary of *P. indusiatus* bioactive compounds and their predicted interactions with inflammatory targets from the STITCH 5.0 protein–chemical network in *Felis catus*. Compounds such as arginine, phenylalanine, tyrosine, cystathionine, sialic acid, mannitol and adenosine demonstrate diverse immunomodulatory activities, including broad immunosuppressive, anti-inflammatory, and antioxidant effects. These interactions highlight their potential roles in attenuating pro-inflammatory cytokines, enhancing IL-10–mediated regulation, and supporting immune balance, comparable to the effects of dexamethasone (positive control) (Table 3).

**Figure 11:**
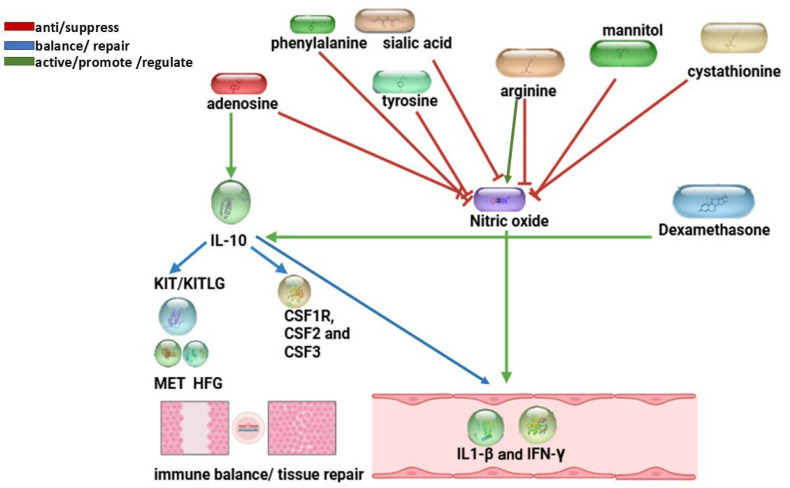
Simplified protein–chemical interaction network generated using STITCH 5.0 for *Felis catus*, integrating *P. indusiatus*–derived bioactive compounds with inflammatory targets and dexamethasone (positive control). Red arrows indicate predicted inhibitory effects on nitric oxide–associated and pro-inflammatory signaling (e.g., IL-1β and IFN-γ), green arrows denote predicted regulatory associations involving IL-10, and blue arrows represent predicted links to colony-stimulating factors and growth factor–related pathways. Color coding is used to visualize predicted interactions.

A simplified summary diagram was subsequently constructed to visualize the principal concentrations between *P. indusiatus* compounds and host inflammatory targets based on LC–MS/MS identification and STITCH 5.0 protein interaction network in *Felis catus*, as shown in Figure 11.

## 3. Discussion

FIP has been reported more frequently in male cats than in females, although sex predisposition is not consistently observed across studies (Moyadee et al., 2019) [49]. In the present study, both the FIP and donor groups showed comparable sex distributions with a predominance of males, suggesting minimal influence of sex on group comparisons. FIP is most commonly diagnosed in young cats, particularly young adults, and cats in both groups in this study were predominantly young adults, resulting in overlapping age distributions that likely reduced age-related biological variability in inflammatory and hematological comparisons.

Hematological abnormalities are commonly observed in cats with FIP; however, they are not consistently present across all cases [2,50]. Cats with effusive FIP demonstrated hematologic patterns compatible with systemic inflammation and a tendency toward mild anemia, while lymphocyte responses showed marked variability. Although the mean lymphocyte count remained within the reference range, lymphopenia was identified in two of eight cats (25%), which is lower than that reported by Yin et al. (2021; 33.9%) and our previous study, in which lymphopenia was detected in up to 75% of affected cats (Moyadee et al., 2024) [50,51]. These findings highlight the heterogeneous nature of hematologic responses in FIP and suggest that lymphocyte depletion is influenced by multiple factors, including disease stage, severity, host immune response, and study population characteristics [50]. Therefore, lymphopenia should be regarded as a variable rather than a consistent hematologic feature of FIP, and its absence does not exclude the diagnosis when interpreted alongside clinical and other supportive diagnostic findings [50,52].

In this study, a concentration of 19 µg/mL was selected for cytotoxicity screening based on previous reports and preliminary tests indicating no cytotoxic effects at this concentration [22]. However, although extract concentrations of 39 and 78 µg/mL yielded cell viability greater than 80% in our assays, these concentrations were not included in subsequent cytokine secretion experiments due to the limited number of PBMCs available from cats. If sufficient cells were available, at least three concentrations should be selected to observe the dose-dependent effects of the extract on cytokine secretion [53,54].

Stimulation of PBMCs from whole blood with 1 µg/mL LPS significantly increased IL-1β, while IL-10 rose modestly (165.39 to 265.58 pg/mL) without significance. This reflects Toll-like receptor 4 (TLR4) mediated monocyte activation, which induces IL-1β but not IFN-γ [55,56]. IFN-γ showed no significant change; this is consistent with the findings of Kanevskiy et al. (2013) [57] and with its primary production by T lymphocytes and NK cells rather than monocytes [58,59]. Notably, PBMCs from FIP fluid exhibited markedly higher IL-1β and IFN-γ levels than LPS-stimulated blood-derived PBMCs, indicating a locally enhanced inflammatory response in FIP cats [60]. Interestingly, IL-10 did not increase in either whole blood or PBMCs from FIP fluid, suggesting insufficient anti-inflammatory feedback. The elevated IFN-γ reflects a Th1-skewed profile associated with macrophage and cytotoxic T-cell activation against intracellular pathogens [61]. These findings indicate that immune cells in FIP fluid are likely pre-activated by viral antigens or inflammatory mediators, sustaining a strong pro-inflammatory environment [11,22].

In PBMCs from whole blood, both dexamethasone and *P. indusiatus* extract significantly suppressed LPS-induced IL-1β, indicating measurable anti-inflammatory activity under normal immune conditions. In contrast, only dexamethasone reduced IL-1β secretion in PBMCs from FIP fluid, consistent with chronic activation driven by persistent viral antigens and inflammatory mediators [62]. IFN-γ displayed a similar pattern: both treatments reduced IFN-γ in PBMCs from fluid, while IFN-γ remained unchanged in LPS-stimulated PBMCs from whole blood, in agreement with Cirkovic Velickovic et al., 2008 [63]. These differences highlight the importance of evaluating immunomodulatory effects within the disease-specific microenvironment rather than under normal immune conditions, in order to ultimately influence the systemic immune response [64]. This context-dependent variation in disease can influence how immune cells respond to stimuli and ultimately shape the overall immunomodulatory outcome [65,66].

In our results, both dexamethasone and *P. indusiatus* extract markedly suppressed IL-10 in PBMCs from whole blood and PBMCs from FIP fluid, with stronger suppression by the extract in FIP fluid-derived cells. Because IL-10 is a key anti-inflammatory cytokine, its reduction may lessen immunosuppressive constraints and enhance antiviral activity, but also risk intensifying inflammation [67]. The parallel suppression of IL-10 in both experimental systems suggests broad modulation of immune activation rather than selective targeting of a single cytokine pathway [39,40,68]. Dexamethasone is known to reduce IL-10 production through inhibition of NF-κB–dependent and receptor-mediated signaling [40,69,70,71]. The simultaneous reduction in pro- and anti-inflammatory cytokines suggests that the extract may target IL-10–producing subsets or upstream nodes enriched in chronically activated effusion-derived cells. Further studies assessing basal cytokine sources, signaling engagement, and viral load are needed to determine whether IL-10 suppression is ultimately protective or detrimental in FIP. Notably, IL-10 modulation in this context likely represents a compensatory or regulatory adjustment of immune signaling in response to chronic inflammation, rather than direct induction of pro-inflammatory activity [72,73] and distinction is important to avoid over-interpretation of IL-10 suppression in the setting of FIP.

Based on our results and previously reported mechanisms (Figure 9), a proposed model illustrates how LPS- and virus-mediated receptor signaling may be modulated by *P. indusiatus* extract and dexamethasone. In LPS-stimulated PBMCs, LPS binds to TLR4 and activates adaptor proteins including mitochondrial antiviral signaling protein (MAVS), NF-κB essential modulator (NEMO), and MAPK branches, including c-Jun N-terminal kinase (JNK) and extracellular signal-regulated kinase (ERK), leading to IκB phosphorylation, NF-κB nuclear translocation, and transcription of IL-1β, NO, and IL-10 [71,74,75]. Downstream of these signals, cytokine-mediated JAK activation may further induce signal transducer and STAT3, which contributes to IL-10 regulation [76]. Both *P. indusiatus* and dexamethasone inhibit this pathway at the receptor-proximal level by suppressing TLR4–NEMO signaling and limiting NF-κB activation (p65/p50). This is consistent with the study by Sedtananun et al. (2025) [77]. In addition, *P. indusiatus* attenuates ERK/MAPK [74,78], which is consistent with the reduced IL-1β and IL-10 secretion observed in LPS-stimulated PBMCs. In PBMCs from FIP fluid, viral RNA recognition through TLR7/8, TLR3, and melanoma differentiation–associated protein (5MDA5) triggers downstream TNF receptor-associated factor (TRAF) adaptors and TRAF2/5 and MAVS, converging on NEMO-mediated activation of NF-κB [79]. In this setting, both treatments may suppress excessive cytokine production by inhibiting MAVS–NEMO–NF-κB signaling, while additional suppression of MAPK and downstream JAK–STAT3 pathways by *P. indusiatus* could account for its stronger inhibition of IL-10 in chronically activated immune cells [80,81]. Together, this model supports the interpretation that *P. indusiatus* extract functions as a broad upstream modulator of pattern-recognition receptor signaling, distinct from but partially overlapping with glucocorticoid-mediated suppression by dexamethasone.

Our in silico predictions aligned with experimental data, illustrating the complex interplay between metabolic substrates, immune receptors, cytokines, and pharmacological modulators that regulate inflammation (Figure 10). The interaction analysis revealed that arginine and adenosine were associated with reduced NO signaling, while arginine simultaneously promoted NOS_2_ and NOS_3_, indicating dual and context-dependent regulatory roles in NO metabolism [31]. Arginine also drives pro-inflammatory signaling that converges on IL-1β, and IFN-γ [30,31]. Although some studies have reported a reduction in nitric oxide production in the presence of arginine-rich extracts, L-arginine is the biological substrate for nitric oxide synthase and is not a direct inhibitor of nitric oxide synthesis [82]. The observed effects on nitric oxide–associated inflammatory responses are therefore more likely to reflect modulation of arginine metabolism or downstream signaling rather than direct inhibition of nitric oxide production [82]. Importantly, cats with FIP are known to exhibit reduced plasma arginine levels, and further depletion of arginine may exacerbate immune dysfunction. In this context, the presence of arginine-related compounds in the extract is more plausibly associated with immune regulatory balance rather than nitric oxide suppression. In the STITCH interaction network, arginine was therefore not interpreted as a blocked or inhibitory node, but rather as a regulatory metabolite potentially involved in the modulation of inflammatory signaling and immune homeostasis.

To integrate the LC–MS/MS and STITCH analyses, a simplified interaction diagram was generated to summarize the principal regulatory relationships between *P. indusiatus*–derived compounds and host inflammatory targets (Figure 11). Several bioactive compounds in *P. indusiatus*, including adenosine, phenylalanine, tyrosine, sialic acid, mannitol, and cystathionine, were predicted to suppress NO production. This is consistent with the study by Theeraraksakul et al. (2023) [83]. Among these, adenosine emerged as a key immunoregulatory mediator, promoting IL-10 production and suppressing pro-inflammatory cytokine signaling through inhibition of the NF-κB pathway [34,35,38,84]. In addition, adenosine interacted with colony-stimulating factors (CSF family members: CSF1R, CSF2, CSF3) and growth factor–related pathways (HGF, KIT/KITLG, MET), linking inflammatory regulation to immune cell differentiation and tissue repair in a manner comparable to the anti-inflammatory profile of dexamethasone (positive control) [41,42,44], LPS appeared as an isolated node in the Felis catus protein–chemical interaction network, indicating no direct interaction with annotated host proteins in this dataset. Collectively, these findings underscore the capacity of *P. indusiatus* extract compounds to restore immune balance by attenuating IL-1β and IFN-γ signaling, enhancing IL-10–mediated regulation, and mitigating oxidative stress, consistent with the LC–MS/MS chemical profile (Table 2). In a broader context, the immunomodulatory effects of *P. indusiatus* extract observed here are comparable to plant-derived phytochemicals such as kaempferol and piperine [85,86]. Kaempferol suppresses pro-inflammatory cytokines via NF-κB and MAPK regulation [86], while piperine modulates cytokine production and immune activation [85]. Similarly, *P. indusiatus* attenuated IL-1β and IFN-γ signaling with low cytotoxicity and context-dependent effects in chronically activated PBMCs from FIP fluid.

Collectively, PBMCs from FIP fluid display a pre-activated inflammatory profile, characterized by elevated IL-1β and IFN-γ and limited anti-inflammatory feedback. The immunomodulatory effects of *P. indusiatus* extract differed from those observed in PBMCs from whole blood, with pronounced suppression of IL-10 in chronically activated cells, suggesting regulation at upstream pattern-recognition receptor pathways. These findings emphasize the context-dependent nature of immune responses in FIP and support the potential of *P. indusiatus* as a multi-target immunomodulator. Overall, this study highlights the importance of evaluating immunomodulatory interventions within disease-specific microenvironments to achieve meaningful therapeutic effects.

## 4. Materials and Methods

### 4.1. Ethics

The study was approved by the Institutional Animal Care and Use Committee of Kasetsart University (Bangkok, Thailand; protocol no. ACKU67-VET-124). All sample collections were conducted with the informed consent of the cat owners.

### 4.2. Animals and Experimental Design

All experimental procedures involving sample collection and diagnosis of feline infectious peritonitis (FIP) were approved by the Kasetsart University Veterinary Teaching Hospital, Bangkok, Thailand. Peripheral blood mononuclear cells (PBMCs) were collected from twenty clinically healthy domestic cats serving as blood donors (*n* = 20). These cats showed no clinical abnormalities and had hematological parameters within established reference ranges.

Pleural or ascitic effusions were obtained from twenty client-owned cats diagnosed with effusive FIP (*n* = 20). The FIP group included cats of various breeds, ages, and sexes (median age: 3.6 months; range: 2–180 months) comprising 11 males and 9 females; detailed signalment and diagnostic information for all cases are provided in Supplementary Table S1. The diagnosis of FIP was established based on a combination of compatible clinical history and signs, characteristic effusion findings, laboratory abnormalities (including a decreased albumin-to-globulin [A:G] ratio < 0.7), positive Rivalta’s test results, cytological evaluation of effusion samples, and molecular detection of feline coronavirus (FCoV) RNA by PCR targeting the 3′ untranslated region (3′UTR). Imaging findings consistent with effusive FIP were also considered, as described in our previous studies [22,87].

Routine blood chemistry profiles were reviewed as part of the clinical assessment but were not included in the present analysis. Due to technical limitations related to PBMC yield and cell quality, only cases yielding sufficient viable PBMCs were included for downstream immunological assays. All selected cases fulfilled the diagnostic criteria described above and were confirmed to be FCoV-positive by PCR. Mononuclear cells isolated from effusion samples are hereafter referred to as “PBMCs from FIP fluid.” The sample size was determined based on our previous work and comparable published reports [20].

### 4.3. The Preparation of the P. indusiatus Extraction

The edible mushroom *P. indusiatus* was selected for further investigation based on our previous screening study, where it exhibited the strongest inhibitory activity against FIPV M^pro^ among 17 medicinal mushrooms tested [22]. In addition, the extract reduced NO production, a marker of inflammation, in LPS-stimulated PBMCs [22]. For extraction, 100 g of air-dried fruiting bodies were finely ground and immersed in 200 mL of 95% ethanol at 37 °C with continuous shaking for 12 h. The mixture was filtered, and the solvent was removed under reduced pressure using a rotary evaporator. The resulting crude extract was stored at −20 °C until further use. All procedures followed the extraction protocol previously described [83].

### 4.4. Preparation of PBMCs and Cell Culture

Peripheral blood was collected from healthy donor cats, and pleural or peritoneal fluid were obtained from FIP cats. PBMCs from whole blood were isolated by density gradient centrifugation using Lymphoprep™ (Corning^®^, Corning, NY, USA) at 600× *g* for 30 min. Cells were resuspended in complete RPMI-1640 medium (Corning^®^, USA) supplemented with 10% fetal bovine serum (FBS; Gibco^®^, Waltham, MA, USA) and 1% penicillin–streptomycin (Gibco^®^, Waltham, MA, USA), and the concentration was adjusted to 1 × 10^6^ cells/mL. Cell counts and viability were assessed using 0.4% Trypan Blue (Gibco^®^, Waltham, MA, USA) with a hemocytometer.

PBMCs from FIP fluid were processed with special consideration for sample viscosity. Highly viscous samples were first diluted with 1× phosphate-buffered saline (PBS; Cytiva HyClone™, Logan, UT, USA) prior to centrifugation at 600× *g* for 10 min. The supernatant was discarded, and the pellet was washed three times with RPMI medium (1:1, *v*/*v*), with each wash followed by centrifugation at 600× *g* for 10 min. The final cell suspensions were counted and cultured under the same conditions as blood-derived PBMCs. The isolation and culture of PBMCs from both whole blood and FIP fluid were performed according to the same standardized protocol [22].

### 4.5. Toxicity of P. indusiatus in PBMCs

PBMCs from whole blood were adjusted to 1 × 10^6^ cells/mL, seeded in 96-well plates, and pre-incubated for 1–2 h in a humidified atmosphere at 37 °C with 5% CO_2_. Cells were treated with *P. indusiatus* extract using a dilution series 19, 39, 78, 156, 625, 2500, 5000 and 10,000 µg/mL in 0.5% DMSO for 24 h. Cell viability was determined using the CCK-8 assay (Dojindo, Kumamoto, Japan) by adding 10 µL of reagent per well, incubating for 2–4 h, and measuring absorbance at 450 nm (EnSight^®^, PerkinElmer, Waltham, MA, USA). Viability was expressed as a percentage relative to untreated controls.

### 4.6. Determination of IL-1β, TNF-γ and IL-10 Release

PBMCs from whole blood (1 × 10^6^ cells/mL) were seeded into 96-well plates and pre-incubated for 1–2 h in a humidified atmosphere with 5% CO_2_ at 37 °C. Cells were then treated with *P. indusiatus* (19 µg/mL) or dexamethasone (5 µg/mL) (Lodexa, Bangkok, Thailand) as a positive control for 2 h, followed by stimulation with LPS (1 µg/mL) and incubation for 24 h. After incubation, supernatants were collected by centrifugation at 3500 rpm for 10 min and stored at −80 °C until analysis. Levels of pro-inflammatory and anti-inflammatory cytokines were quantified using commercial IL-1β, IFN-γ, and IL-10 ELISA kits (Abcam, Waltham, MA, USA). A parallel test was conducted on PBMCs from FIP fluid under the same conditions but without LPS stimulation.

### 4.7. Extraction and LC–MS/MS Characterization of Bioactive Compounds in P. indusiatus

Fifty milligrams of *P. indusiatus* were extracted in 1 mL of 50% ethanol. The extracts were incubated at 50 °C for 1 h, with vertexing for 10 min during incubation. Samples were centrifuged at 12,000 *g* for 10 min, and the supernatant was transferred to a new tube. The extraction was repeated twice, and the supernatants were combined prior to lyophilization. The dried extracts were reconstituted in 1 mL of 50% ethanol and further diluted 1:10 in LC–MS/MS grade solvent. Before injection, the extracts were filtered through a 0.22 µm PTFE membrane.

LC–MS/MS analysis was performed using a Thermo Q Exactive™ HF-X Orbitrap mass spectrometer (Thermo Fisher Scientific, Bremen, Germany) coupled to a Vanquish™ Horizon UHPLC system (Thermo Fisher Scientific, Waltham, MA, USA). Separation was achieved on an Accucore™ Vanquish C18 column (2.1 × 100 mm, 1.5 µm; Thermo Scientific, Waltham, MA, USA) with a guard column at 40 °C. Sample injections of 0.2 µL were run at 0.2 mL/min. The mobile phase consisted of water with 0.1% formic acid (A) and acetonitrile with 0.1% formic acid (B). The gradient started at 90% A and 10% B, held for 3 min, increased to 90% B over 10 min, flushed at 90% B for 3 min, then returned to starting conditions over 0.5 min; total run time was 20 min. Mass spectrometry was conducted in both positive and negative ESI modes using full-scan MS1 and data-dependent MS2 (dd-MS2). The scan range was 150–2000 *m*/*z*, with ion fragmentation performed using stepped normalized collision energies (NCE) of 20, 30, and 40 eV. The interaction network of mechanisms of action, a protein–chemical interaction network was generated using STITCH 5.0 for the *Felis catus* organism (http://stitch.embl.de/) (accessed on 30 August 2025) [87,88,89].

### 4.8. Statistical Analysis

Hematological parameters between donor cats and FIP cats were analyzed using two-sample *t*-test, with *p* < 0.05 considered statistically significant. PBMCs viability and cytokine levels after LPS stimulation were analyzed using one-way ANOVA, while comparison of cytokine levels between blood-derived and fluid-derived PBMCs were compared using two-way ANOVA. In all analyses, *p* < 0.05 was considered statistically significant. Data are presented as mean ± standard error of the mean (SEM), and each experiment was replicated at least twice to ensure reliability. All analyses were conducted using GraphPad Prism version 9 (GraphPad Software, LLC, Boston, MA, USA).

## 5. Conclusions

This study demonstrates that *P. indusiatus* extract exerts immunomodulatory activity in feline PBMCs derived from both whole blood and FIP fluid, as evidenced by the reduction in key pro-inflammatory cytokines (IL-1β and IFN-γ) and context-dependent modulation of IL-10. The observed cytokine patterns, which were comparable to those induced by dexamethasone, are consistent with modulation of upstream inflammatory signaling pathways previously implicated in cytokine regulation, including TLR-, MAPK-, NF-κB-, and JAK–STAT-associated pathways. PBMCs derived from FIP fluid exhibited strong basal activation and a distinct cytokine profile, highlighting the importance of evaluating immunomodulatory agents within disease-specific immune microenvironments. LC–MS/MS profiling and STITCH network analysis identified bioactive compounds such as adenosine, phenylalanine, tyrosine, cystathionine, and N-acetylated sugars that have been previously reported to possess anti-inflammatory, antioxidant, and immune-regulatory properties. Collectively, these findings support the potential of *P. indusiatus* as a natural immunomodulatory candidate for FIP and provide a rationale for future mechanistic and in vivo studies to validate the signaling pathways involved.

## Figures and Tables

**Table 1:** Establishing a hematological profile for early detection of feline infectious peritonitis.

Parameter	Normal Reference	Healthy Donor Cats (*n* = 8)				FIP-Infected Cats (*n* = 8)				*p*-Value <0.05
		mean	min	max	median	mean	min	max	median	
HGB (mg/dL)	9.8–15.4	13.80	11.50	15.50	14.30	10.03	5.70	15.00	10.10	0.0069 *
PCV %	30–45	42.10	34.70	47.80	43.40	27.52	16.70	41.60	26.60	0.0003 *
RBC (×10^6^/µL)	5.0–10.0	8.85	8.16	9.93	8.79	6.21	2.89	10.63	6.66	0.0098 *
WBC (×10^3^/µL)	5.5–19.5	9.46	6.40	12.34	9.39	27.91	4.85	68.79	15.55	0.0321 *
SEGS% (×10^3^/µL)	2.5–12.5	9.46	6.40	12.34	9.39	5.30	2.23	7.10	5.70	0.0002 *
LYMPH% (×10^3^/µL)	1.5–7	3.28	1.71	4.52	3.76	3.88	0.44	11.01	3.31	0.9795
MONO% (×10^3^/µL)	0–0.9	0.22	0.00	0.56	0.16	0.90	0.00	3.44	0.45	0.0953
EOS% (×10^3^/µL)	0–0.8	0.55	0.00	1.60	0.56	1.04	0.00	3.44	0.52	0.2575
RETICS (×10^6^/µL)	0–0.6	9.46	6.40	12.34	9.39	5.30	2.23	7.10	5.07	0.0002 *
PLATELETS (×10^3^/µL)	200–800	206.89	92.00	363.00	216.00	251.50	27.00	372.00	327.50	0.4074

Data are presented as mean, minimum, maximum, and median values. Donor cats (*n* = 8) and FIP cats (*n* = 8) were included in the analysis. HGB, hemoglobin; PCV, packed cell volume; RBC, red blood cell count; WBCs, white blood cell counts; Segs, segmented neutrophils; LYMPH, lymphocytes; MONO, monocytes; EOS, eosinophils; RETICS, reticulocytes. * *p* < 0.05 compared with donor cats.

**Table 2:** LC–MS/MS identification of chemical constituents in *P. indusiatus* extract.

No.	Tentative Identification Name	RT (min)	Molecular Weight	[M + H]^+^ (*m*/*z*)	[M-H]^−^ (*m*/*z*)	Formula	PubChem CID
1	N-(1-Deoxy-1-fructosyl)valine	1.992	279.1319	280.1391	-	C_11_H_21_NO_7_	131,752,247
2	N-(1-Deoxy-1-fructosyl)leucine	2.156	293.1475	294.1547	-	C_12_H_23_NO_7_	131,752,244
3	1-(3-Amino-3-carboxypropyl)-3-pyridiniumcarboxylate	1.900	224.0796	225.0870	-	C_10_H_12_N_2_O_4_	-
4	1-[(3-Carboxypropyl)amino]-1-deoxy-beta-D-fructofuranose	1.918	265.1162	266.1234	-	C_10_H_19_NO_7_	131,752,417
5	1-linoleoyl-sn-glycero-3-phosphoethanolamine	13.004	477.2857	478.2928	-	C_23_H_44_NO_7_P	52,925,130
6	2-Acetamido-2-deoxyglucose	1.939	221.08951	-	220.0827	C_8_H_15_NO_6_	343,911
7	2-Oxo-5-pentyltetrahydro-3-furancarboxylic acid	9.592	200.10415	-	199.0976	C_10_H_16_O_4_	-
8	3-O-beta-D-galactosyl-sn-glycerol	2.044	254.09977	-	253.0929	C_9_H_18_O_8_	16,048,618
9	Adenosine	2.028	267.09684	268.1040	-	C_10_H_13_N_5_O_4_	60,961
10	Argininosuccinic acid	1.895	290.1227	291.1299	-	C_10_H_18_N_4_O_6_	16,950
11	Cystathionine	1.879	222.06703	223.0747	221.0605	C_7_H_14_N_2_O_4_S	834
12	D-(-)-Mannitol	1.910	182.07822	-	181.0718	C_6_H_14_O_6_	6251
13	D-(+)-Glucose	1.919	180.0627	-	179.0561	C_6_H_12_O_6_	107,526
14	DL-Arginine	1.853	174.11155	175.1189		C_6_H_14_N_4_O_2_	232
15	DL-Tyrosine	2.154	181.07381	182.0812	-	C_9_H_11_NO_3_	1153
16	Gluconic acid	1.936	196.05753	-	195.0510	C_6_H_12_O_7_	10,690
17	Hercynine	1.869	197.11634	198.1237	-	C_9_H_15_N_3_O_2_	5,459,798
18	Hexitol	1.933	182.0789	183.0863	-	C_6_H_14_O_6_	453
19	L-Histidine	1.852	155.06936	156.0767	-	C_6_H_9_N_3_O_2_	6274
20	L-Iditol	1.905	182.07825	-	181.0718	C_6_H_14_O_6_	5,460,044
21	L-Phenylalanine	2.496	165.07875	166.0863	-	C_9_H_11_NO_2_	6140
22	L-Tyrosine	2.086	181.07373	182.0812	-	C_9_H_11_NO_3_	1153
23	Muramic acid	1.933	251.10055	252.1078	-	C_9_H_17_NO_7_	441,038
24	N-Acetyl-D-galactosamine	1.928	267.09508	-	266.0881 *	C_8_H_15_NO_6_	35,717
25	N-Acetyl-α-D-glucosamine	1.951	221.08987	222.0972	-	C_8_H_15_NO_6_	24,139
26	N-Acetylneuraminic acid	1.945	309.106	310.1133	-	C_11_H_19_NO_9_	439,197
27	N(2)-Acetyl-L-ornithine	1.938	174.10046	175.1077	-	C_7_H_14_N_2_O_3_	439,232
28	α-Lactose	1.928	359.14285	360.1500 **		C_12_H_22_O_11_	84,571

* [M-H + HCOOH]^−^ adduct, ** [M + NH4]^+^ adduct.

**Table 3:** Predicted inflammatory effects of *P. indusiatus* bioactive compounds and associated immune-related targets in cats, based on STITCH analysis.

Compound/Substrate	Effect on Inflammation	References
Arginine	Fuels NO production; drives pro-inflammatory signaling (TNF, IL-1, IFN-γ)	[30,31]
Phenylalanine, Tyrosine, Cystathionine	Associated with modulation of arginine-dependent and NO-related inflammatory pathways, suggesting a regulatory role in limiting excessive inflammatory response	[32,33]
Adenosine	Suppresses cytokine release (IL-8, IL-1β, and IFN-γ) while enhancing IL-10	[34,35,36,37,38]
IL-10	Central regulator; counterbalances pro-inflammatory cascades	[39,40]
CSF family (CSF1R, CSF2, CSF3)	Link inflammation to immune cell differentiation and repair	[41,42]
Growth factors (HGF, KIT/KITLG, MET)	Context-dependent roles in promoting/resolving inflammation	[41,42,43]
GalNAc, NAG	Reported anti-inflammatory and antiviral properties (no direct links)	[21,22]
Dexamethasone (positive control)	Potent immunosuppressive; suppress or enhance IL-10 production.	[44,45]
Sialic acid	The mechanisms include inhibiting leukocyte accumulation, regulating immune cell signaling pathways such as MAPK–NF-κB/AP-1, and affecting the activity of antibodies such as IgG.	[46]
Mannitol	The anti-inflammatory mechanism involves inhibiting NF-κB activation, suppressing lipid peroxidation, and reducing oxidative stress	[47,48]

## Data Availability

The raw data supporting the conclusions of this article will be made available by the authors on request. Further inquiries can be directed to the corresponding author.

## Supplementary Materials

The following supporting information can be downloaded at: https://www.mdpi.com/article/10.3390/ijms27031437/s1.
