# Supplementary material for: Immunomodulatory Effects of Phallus indusiatus Extract on Cytokine Responses in PBMCs: Implications for Feline Infectious Peritonitis
Source: Int J Mol Sci. 2026 Jan 31;27(3):1437. doi: 10.3390/ijms27031437 (PMC12898788; doi:10.3390/ijms27031437)
Supplement: Supplementary file 1 [file ijms-27-01437-s001.zip › ijms-4097979-supplementary.pdf]

Table S1. Clinical and laboratory findings of cats suspected of feline infectious peritonitis (FIP).

No.	Sex	Age (years)	Color of fluid	A: G Ratio	PCR	Rivalta's test
**1**	female	8.8	yellow	0.15	positive	positive
**2**	male	4.5	yellow	2	negative	positive
**3**	female	3.3	yellow	4	negative	positive
**4**	male	1.3	yellow	0.3	positive	positive
**5**	male	0.2	yellow	0.2	positive	positive
**6**	female	0.7	yellow	2.5	negative	positive
**7**	male	4	yellow	4	negative	positive
**8**	female	9	yellow	1	positive	positive
**9**	male	1.2	yellow	0.1	positive	positive
**10**	male	2.5	yellow	1.3	negative	positive
**11**	female	12	yellow	1.8	negative	positive
**12**	male	2.1	yellow	0.2	positive	positive
**13**	male	0.6	yellow	0.5	positive	positive
**14**	female	13.3	yellow	0.3	positive	positive
**15**	male	15	yellow	5	positive	positive
**16**	male	14.9	yellow	6	positive	positive
**17**	female	0.7	yellow	0.2	positive	positive
**18**	Female	5	yellow	5	positive	positive
**19**	female	6	yellow	6	positive	positive
**20**	male	0.7	yellow	0.4	negative	positive
